# Correction: Uncovering diverse reactivity of NHCs with diazoalkane: C–H activation, C=C bond formation, and access to N-heterocyclic methylenehydrazine

**DOI:** 10.1039/d5sc90098k

**Published:** 2025-05-21

**Authors:** Kajal Balayan, Himanshu Sharma, Kumar Vanka, Rajesh G. Gonnade, Sakya S. Sen

**Affiliations:** a Inorganic Chemistry and Catalysis Division, CSIR-National Chemical Laboratory Dr Homi Bhabha Road, Pashan Pune 411008 India ss.sen@ncl.res.in; b Academy of Scientific and Innovative Research (AcSIR) New Ghaziabad 201002 India; c Physical and Material Chemistry Division, CSIR-National Chemical Laboratory Dr Homi Bhabha Road, Pashan Pune 411008 India rg.gonnade@ncl.res.in

## Abstract

Correction for ‘Uncovering diverse reactivity of NHCs with diazoalkane: C–H activation, C=C bond formation, and access to N-heterocyclic methylenehydrazine’ by Kajal Balayan *et al.*, *Chem. Sci.*, 2024, **15**, 18387–18394, https://doi.org/10.1039/D4SC05740F.

The authors regret that there is an error in the previously published version of Fig. 12. The structure was inadvertently labelled as COOEt, whereas it should be COO^*t*^Bu. However, all calculations were performed using COO^*t*^Bu, so the reported energy values remain unchanged. The corrected scheme is shown below. This correction does not affect any of the conclusions presented in the work.

**Fig. 12:**
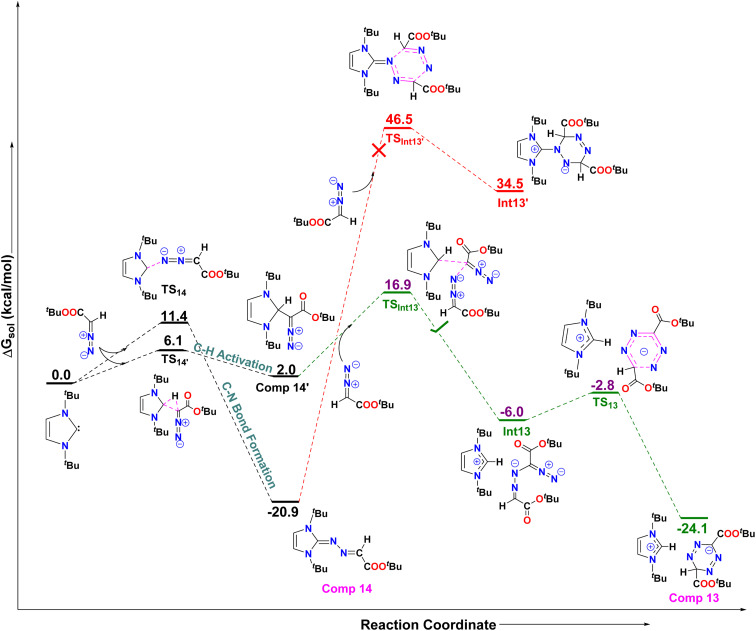
The free energy profiles for the formation of compound **13** and compound **14** have been shown here. All values are in kcal mol^−1^. Level of theory: PBE0-D3/def2TZVP//PBE-D3/defTZVP with solvent toluene.

The Royal Society of Chemistry apologises for these errors and any consequent inconvenience to authors and readers.

